# Malnutrition and lipid abnormalities in antiretroviral naïve HIV-infected adults in Addis Ababa: A cross-sectional study

**DOI:** 10.1371/journal.pone.0195942

**Published:** 2018-04-19

**Authors:** Melaku Adal, Rawleigh Howe, Desta Kassa, Abraham Aseffa, Beyene Petros

**Affiliations:** 1 Microbial, Cellular and Molecular Biology Department, Addis Ababa University, Addis Ababa, Ethiopia; 2 Armauer Hansen Research Institute, Addis Ababa, Ethiopia; 3 Ethiopian Public Health Institute, Addis Ababa, Ethiopia; University of New South Wales, AUSTRALIA

## Abstract

**Background:**

Both under- and over-nutrition may occur among human immunodeficiency virus (HIV)-infected individuals and impact on the course of the acquired immune deficiency syndrome (AIDS) and its management due to the close interaction between immunity and nutrition. We investigated occurrence of undernutrition, excess weight and lipid abnormalities among antiretroviral naïve HIV-infected adults in Addis Ababa, Ethiopia.

**Methods:**

A cross-sectional study on 594 antiretroviral therapy (ART) naïve HIV-infected adults was conducted in four hospitals in Addis Ababa from February to September 2013. Hematological parameters (CD4+ T cell count and hemoglobin concentration), fasting serum glucose, total cholesterol (TC) and triglycerides (TG) were determined. Information on socio-demographic, anthropometric and World Health Organization (WHO) clinical stages was collected from patient clinical records, and triangulated by structured questionnaire. Height and weight measurements were taken and body mass index (BMI), undernutrition (BMI <18.5 kg/m^2^) and excess weight (BMI ≥25 kg/m^2^) determined. Statistical comparisons were made to identify significant factors associated with nutritional status and lipid profiles.

**Results:**

The prevalence of undernutrition was 15.1%, and the prevalence of excess weight was 22.1%, including 5.4% who were obese. The prevalence of hypercholesterolemia was 16.6% and it was higher in women (18.9%) than in men (11.0%) (p<0.05). However, the prevalence of hypertriglyceridemia was 29.8%. There was significant positive Spearman correlation between CD4+ T cell count and serum TC (r = 0.210, p<0.001), but no correlation was observed between CD4+ T cell count and TG (r = -0.007, p>0.05). Age categories 30–39 and 40–79, and WHO clinical stages III/IV for undernutrition; age categories 30–39 and 40–79, WHO clinical stages III/IV and TC ≥200 mg/dL for excess weight; and being female, age categories 30–39 and 40–79, and hypertriglyceridemia for hypercholesterolemia were found to be independent predictors by binomial logistic regression analysis.

**Conclusion:**

Undernutrition, excess weight, hypercholesterolemia and hypertriglyceridemia were variably prevalent in ART naïve HIV-infected populations. This emphasizes the need for targeted nutritional programs as an integral part of HIV/AIDS care. Lipid levels need to be monitored regularly in patients whether on or off ART. In addition, improvement on household income and positive change in lifestyle and/or nutritional treatment to reduce morbidity and mortality are necessary interventions in HIV/AIDs patient management.

## Introduction

Poor nutritional status can impair immunity independent of HIV infection, and impaired immunity increases susceptibility of individuals to infections [1]. HIV weakens the immune system and impairs nutritional status through the reduction of nutrient intake due to nausea, vomiting, abdominal pain, anorexia and HIV-induced enteropathy. The nutritional status could be compromised due to food insecurity, lack of food safety, lack of hygienic care and dehydration due to diarrhea. In addition, maldigestion and malabsorption due to infection, liver and kidney disease leading to altered use, impaired storage and excretion of nutrients, increased requirements for both macro- and micro-nutrients and medication-related side effects are also factors that affect nutritional status [2–4]. Undernutrition in HIV-infected individuals has been shown to be associated with increased susceptibility to AIDS-related illnesses and disease progression, higher mortality rates and suboptimal response to HIV drugs [5, 6]. Excess weight contributes to health-related metabolic complications such as dyslipidemia, insulin resistance, diabetes, hypertension, lipodystrophy and cardiovascular diseases (CVDs) [7–9]. Increased dyslipidemia in HIV infection increases the risk of atherosclerosis and CVDs [10, 11].

Dyslipidemia is common in HIV patients [12–16]. HIV-related dyslipidemia could be caused by impaired reverse cholesterol transport (RCT) by HIV nef protein that causes degradation of adenosine triphosphate (ATP) binding cassette subfamily A member 1 (ABCA1), impaired peripheral free fatty acid (FFA) trapping in adipose tissue, increased FFAs influx to the circulation that cause abnormal signaling mediated by increased inflammatory cytokines that impaired clearance of TG from the circulation due to reduced lipase activity, increased apolipoprotein B (APOB) and inhibition of its degradation, increased low density lipoprotein cholesterol (LDL-C) and very low density lipoprotein cholesterol (VLDL-C), decreased high density lipoprotein cholesterol (HDL-C), and increased oxidative stress and lipid peroxidation [17–19].

The increased VLDL/LDL possesses a greater content of apolipoprotein C3 (APOC3) that result in reduced lipoprotein lipase activity, and consequently less TG hydrolysis and increased cholesterylester transport protein (CETP) activity that exchanges cholesterylesters from HDL for TG from VLDL/LDL [20, 21]. Then, the stimulation of lipoprotein lipase and phospholipase A2 occurs by the production of HDL with high TG content that results in much smaller HDL with a reduced affinity for apolipoprotein A1 (APOA1). This leads to its dissociation from HDL and subjected to renal excretion due to their small sizes and to lower levels of HDL-C in circulation [22]. In addition, the remnants of VLDL/LDL are rich in cholesterol esters and poorly recognized by both LDL receptor (LDLR) and low density receptor related protein (LRP) for their delayed clearance from the circulation and may result in increased atherosclerosis and CVD risk in HIV-positive individuals [23, 24].

Furthermore, dyslipidemia does not develop in every HIV-positive individual and on those who takes the same ART regimens, suggesting that host factors such as genetics and immunology could play a major role in their development [14, 25]. Candidate genes as well as genome-wide-based association studies have identified single nucleotide polymorphisms (SNPs) that could account for a significant portion of the variation and modulation in blood lipid levels [26–28].

Non-infectious health disorders and nutritional impairment have been increasing in sub-Saharan Africa because of socio-economic changes which are taking place due to urbanization and changing lifestyles [8, 29]. A number of studies from developed world have documented a high rate of dyslipidemia in HIV-infected individuals both on and off ART [30]. Few studies from resource limited settings have shown low-to-normal TC and LDL-C, elevated TG and decreased HDL-C among ART-naïve individuals [31, 32]. Even if there is information on undernutrition, excess weight and lipid abnormalities from other parts of the world, this cannot be extrapolated to the Ethiopian context due to socio-demographic, economic and genetic differences between populations. Therefore, this cross-sectional study was conducted to explore the prevalence of malnutrition and lipid abnormalities, and to identify factors associated with these abnormalities in ART naïve HIV-infected individuals in Addis Ababa, Ethiopia.

## Materials and methods

### Study population, setting and design

This cross-sectional study was conducted as part of a larger study that investigates the effect of viral sequence diversity on the immune response of HIV infected ART naïve patients in Ethiopia. The study participants were HIV-infected adults of both sexes aged ≥18 years who were being followed and newly enrolled at All African Leprosy Rehabilitation and Training Centre (ALERT), St. Paul, Yekatit-12 and Zewditu Memorial Hospitals between February to August 2013 but had not received ART yet. They were consecutively enrolled upon informed consent by anti-retroviral treatment nurses under close supervision of the lead investigator (MA). Patients with severe cognitive impairment, those who are severely morbid or under intensive care, pregnant women and those taking medication that affects serum lipid levels were not included.

### Haematological and biochemical assays

Blood samples were collected using ethylene-diamine-tetraacetic acid (EDTA) vacutainer tubes to determine CD4+ cells count and hemoglobin concentrations. CD4+ T cell count were determined by using automated FACS counter (Becton and Dickinson, San Jose, CA, USA) and categorized according to clinical significance as CD4+ T cell count <200 cells/mm^3^ and those with ≥200 cells/mm^3^. Hemoglobin concentration was determined by using Sysmex-21 automated blood analyzer by noncyanide method (Sysmex, KX-21N, Kobe, Japan). Fasting TC, TG and glucose levels were determined by enzymatic colorimetric method (Human diagnostics, HumanStar 180, Wiesbaden, Germany). Those study subjects with hemoglobin concentration <12 g/dL to women and <13 g/dL to men were considered anemic [33]. Lipid and glucose levels were determined from serum. Metabolic abnormalities were defined according to the National Cholesterol Education Program (NCEP) Adult Treatment Panel III (ATP III) criteria of USA [34]. Hypercholesterolemia was defined by serum TC≥200 mg/dL and hypertriglycerdemia was defined by serum TG≥150 mg/dL. In addition, hyperglycemia was defined when fasting glucose level >110 mg/dL, and impaired fasting glucose (IFG) and diabetes were defined by the fasting serum glucose levels 110-125- and ≥126 mg/dL, respectively.

### Questionnaire

Socio-demographic and WHO clinical stage data were collected from patients’ medical charts and triangulated by means of the data collected by additional structured questionnaire (S1 Table). The questionnaire is validated through expert comments and pilot study from 20 individuals and modified accordingly before the start of the study.

### Anthropometric measurements

Weight to the nearest 100 gram and height to the nearest 1 millimeter were measured. BMI is defined as individual’s body weight divided by the square of their height (kg/m^2^). The protein-energy nutritional status of each study subject was determined using WHO-established BMI cut offs [thinness or acute undernutrition (BMI <18.5 kg/m^2^), normal (BMI = 18.5–24.9 kg/m^2^), overweight (BMI = 25–29.9 kg/m^2^) and obese (BMI ≥30kg/m^2^). Excess weight is defined as BMI ≥25 kg/m^2^ [35].

### Data analysis

Questionnaire and laboratory data were analyzed using STATA version 11.0 (Stata Corp, College station, Texas, USA) and GraphPad Prism version 5.03 (GraphPad software, California, USA). The descriptive information was presented as median values with interquartile range (IQR), frequency counts and percentages. Categorical data were analyzed by Pearson chi-square to test the associations and Fisher’s exact test were applied whenever expected values are lower than 5 for more than 20% of the cells. Pearson chi-square for trend was also done. The Mann-Whitney and Kruskal-Wallis tests were used to compare variables with non-normal distribution. Spearman correlation of serum TC and TG levels with CD4+ T cell count were done. Independent variables which were identified as statistically significant in the univariate analysis (for crude odds ratio, COR) were subsequently used for multivariate logistic regression analysis (for adjusted odds ratio, AOR) for adjusting potential confounders. Confidence interval (CI) of 95% with level of significance α <0.05 was considered to determine the precision of the study.

### Ethical consideration

The study was ethically cleared by the Institutional Research Ethics Review Committees (IRERCs) of participating Institutions and the National Ethical Review Committee, Ministry of Science and Technology with renewed ethical approval reference number 3.10/004/2015 (S1 Fig). A written consent was obtained from study participants.

## Results

### Characteristics of the study population

Among these ART naïve study participants, 423 (71.2%) were women (Table 1). About 74% of the study participants stay positive for more than a year knowing their HIV-positive serostatus. The median age of the study participants who were included in the study was 34 (IQR = 32.0–35.0) years. The median ages of men and women were 37 and 32, respectively, where women were younger (p<0.001). Seventy nine percent of the study participants were with or above primary level of education; and 69.9% were earning <2 dollars per day. About 83% of the study participants had CD4+ T cell count ≥200 cells/mm^3^ with median of 357 cells/mm^3^ (IQR = 248–537). Clinically, 14.4% were at WHO clinical stages III/IV. The total proportion of study participants at AIDS stage with CD4+ T cell count <200 cells/mm^3^ or WHO clinical stages III/IV were 25.9%. The nutritional status of the study participants was found 5.4% obese, 16.7% overweight and 15.1% undernourished. The random fasting glucose level showed that the overall hyperglycemia in the study population was 13.6%. Among which 7.5% were with impaired fasting glucose (IFG) state and 6.1% were diabetic. In addition, the prevalence of anemia was 11.2% in the overall study population, and it was 12.8% in men and 10.6% in women with no significant difference between the two sexes (p>0.05).

**Table 1:** Population characteristics among HIV-infected ART naïve study participants in Addis Ababa, Ethiopia.

	Variables	Number	% (95% CI)
Sex			
	Male	171	28.8 (22.0–35.6)
	Female	423	71.2 (66.9–75.5)
Age			
	18–29	170	28.7 (21.9–35.5)
	30–39	266	44.7 (38.7–50.7)
	40–79	157	26.5 (19.6–33.4)
Education			
	no formal	124	21.0 (13.8–28.2)
	Primary	219	37.1 (30.7–43.5)
	Secondary	188	31.8 (25.1–38.5)
	Tertiary	60	10.1 (2.5–17.7)
Income per day (dollar)			
	<1	162	34.5 (27.2–41.8)
	1–2	171	36.4 (29.2–43.6)
	>2	137	29.1 (21.5–36.7)
Length of time stay positive (years)			
	<1	150	26.1 (19.1–33.1)
	1–3	177	30.8 (23.8–37.4)
	>3	248	43.1 (37.1–49.5)
BMI (kg/m^2^)			
	<18.5	87	15.1 (7.6–22.6)
	18.5–24.9	362	62.8 (57.8–67.8)
	≥25.0	127	22.1 (14.9–29.3)
WHO clinical stage			
	Stage 1	328	55.6 (50.2–61.0)
	Stage 2	177	30.0 (23.2–36.8)
	Stage ¾	85	14.4 (6.9–21.9)
CD4+ cell count (cells/mm^3^)			
	<200	102	17.2 (9.9–24.5)
	≥200	492	82.8 (79.5–86.1)
Hemoglobin level (g/dL)			
	Non-anemic	507	88.8 (86.1–91.5)
	Anemic	64	11.2 (3.5–18.9)
Fasting glucose conc. (mg/dL)			
	Normal (<110)	413	84.4 (80.9–87.9)
	IFG (110–125)	36	7.5 (-1.1–16.1)
	Diabetic (≥126)	29	6.1 (-2.6–14.8)
TC (mg/dL)			
	<200	472	83.3 (79.9–86.7)
	≥200	95	16.7 (9.2–24.2)
TG (mg/dL)			
	<150	401	70.2 (65.7–74.7)
	≥150	170	29.8 (22.9–36.7)

TC = total cholesterol; IFG = impaired fasting glucose; TG = triglycerides; CI = confidence interval; mg/dL = milligram per deciliter; g/dL = gram per deciliter; cells/mm^3^ = cells in cubic millimeter; kg/m^2^ = kilogram per meter square.

### Prevalences of undernutrition and excess weight

The overall median BMI was 21.9 (IQR = 21.5–22.2). It was found to be 21.5 in men and 21.9 in women study participants. The prevalence of undernutrition was 15.1% (95% CI: 7.6–22.6) in overall, 15.8% in men and 14.8% (95% CI: 5.9–23.7) in women. The prevalence of excess weight (overweight or obese) was 22.1% (95% CI: 11.9–31.4) that includes 16.7% (95% CI: 9.2–24.2) overweight and 5.4% (95% CI: -2.6–13.4) obesity in the overall study participants. Excess weight was 17.6% in men and 23.8% in women. On the otherhand, the prevalence of obesity was 3.6% in men and 6.1% in women study participants. The median BMI, the prevalences of undernutrition, excess weight and obesity were not significantly different between men and women (p>0.05).

### Factors associated with undernutrition and excess weight

The association of all independent variables with dependent variables (undernutrition and excess weight) was analyzed by chi-square test (Table 2) and univariate binomial logistic regression analysis (Table 3). Variables that have significant association by chi-square test and/or univariate analysis (age, WHO clinical stages and being anemic/normal for undernutrition; and age, education, income, WHO clinical stages, and hypercholesterolemia/normal for excess weight) were considered to multivariate binomial logistic regression analysis (p<0.05).

**Table 2:** Chi-square associations of risk factors with malnutrition among HIV-infected ART naïve study participants in Addis Ababa, Ethiopia.

Variables		Undernutrition (BMI<18.5)		Excess weight (BMI ≥25)	
		<18.5	≥1.8.5	≥25	<25
		N (%)	N (%)	N (%)	N (%)
Age					
	18–29	35(40.2)	129(26.4)^c^	17(13.5)	147(32.7)^a^
	30–39	38(43.7)	221(45.3)	62(49.2)	197(43.9)
	40–79	14(16.1)	138(28.3)	47(37.3)	105(23.4)
Education					
	no formal	19(21.8)	97(20.0)	12(9.4)	104(23.3)^b^
	Primary	35(40.2)	177(36.4)	43(33.9)	169(37.9)
	Secondary	25(28.7)	161(33.1)	54(42.5)	132(29.6)
	Tertiary	8(9.2)	51(10.5)	18(14.2)	41(9.2)
Income per day (dollar)					
	<1	24(35.3)	132(33.9)	23(23.9)	133(36.8)^c^
	1–2	28(41.2)	139(35.7)	35(36.5)	132(36.6)
	>2	16(23.5)	118(30.3	38(39.6)	96(26.6)
WHO clinical stages					
	Stage I	35(40.7)	284(58.3)^a^	88(69.3)	231(51.8)^c^
	Stage II	25(29.1)	147(30.2)	30(23.6)	142(31.8)
	Stage III/IV	26(30.2)	56(11.5)	9(7.1)	73(16.4)
Hemoglobin level (g/dL)					
	Non-anemic	71(81.6)	424(91.0)^b^	111(94.1)	384(88.3)
	Anemic	16(18.4)	42(9.0)	7(5.9)	51(11.7)
TC (mg/dL)					
	<200	73(88.0)	386(82.1)	81(67.5)	374(87.2)^a^
	≥200	10(12.0)	84(17.9)	39(32.5)	55(12.8)
Fasting glucose conc. (mg/dL)					
	<126	64(98.5)	375(93.3)	90(89.1)	349(95.4)^b^
	≥126	1(1.5)	27(6.7)	11(10.9)	17(4.6)

Note

a, b, c refers p value <0.001, <0.01 and <0.05, respectively. TC = total cholesterol; N = number of study participants with outcome variables by risk factors; % = percentage.

**Table 3:** Univariate and multivariate associations of risk factors with malnutrition among HIV-infected ART-naïve study participants in Addis Ababa, Ethiopia.

Variables		Undernutrition (BMI<18.5)		Excess weight (BMI≥25)	
		COR(95% CI)	AOR(95% CI)	COR(95% CI)	AOR(95% CI)
Age					
	18–29	1.00	1.00	1.00	1.00
	30–39	0.63(0.38–0.99)^b^	0.54(0.32–0.91)^c^	2.72(1.53–4.85)^b^	3.23(1.38–7.55)^b^
	40–79	0.37(0.19–0.73)^b^	0.35(0.18–0.70)^b^	3.30(1.80–6.04)^a^	3.64(1.47–8.98)^b^
Education					
	no formal	1.00	-	1.00	1.00
	Primary	1.01(0.55–1.86)		2.21(1.11–4.37)^c^	1.73(0.65–4.60)
	Secondary	0.79(0.41–1.51)		3.55(1.80–6.97)^a^	2.29(0.83–6.31)
	Tertiary	0.80(0.33–1.96		3.80(1.68–8.60)^b^	2.24(0.66–7.64)
Income per day (dollar)					
	<1	1.00		1.00	1.00
	1–2	1.11(0.61–2.00)	-	1.53(0.86–2.73)	1.38(0.64–2.94)
	>2	0.75(0.38–1.47)		2.29(1.28–4.09)^b^	1.92(0.85–4.33)
WHO clinical stages					
	Stage I	1.00	1.00	1.00	1.00
	Stage II	1.38(0.80–2.39)	1.35(0.77–2.37)	0.55(0.35–0.88)^c^	0.63(0.33–1.22)
	Stage III/IV	3.77(2.10–6.75)^a^	3.54(1.92–6.51)^a^	0.32(0.16–0.67)^b^	0.32(0.10–0.98)^c^
Hemoglobin level (g/dL)					-
	Non-anemic	1.00	1.00	1.00	
	Anemic	2.27(1.21–4.26)^c^	1.84(0.94–3.58)	0.47(0.21–1.08)	
TC (mg/dL)					
	<200	1.00	-	1.00	1.00
	≥200	0.63(0.31–1.27)		3.27(2.04–5.27)^a^	3.97(2.03–7.77)^a^
Fasting glucose conc. (mg/dL)					
	<126	1.00	-	1.00	1.00
	≥126)	0.20(0.03–1.62)		2.51(1.14(5.55)^c^	2.07(0.78–5.51)

Note

a, b, c refers p value <0.001, <0.01 and <0.05, respectively. BMI = body mass index; CI = confidence interval; COR = crude odds ratio; AOR = adjusted odds ratio; TC = total cholesterol. Only variables which have significant association with the outcome variable in univariate analysis are considered for multivariate analysis.

Age and WHO clinical stages III/IV were found to be associated with undernutrition (p<0.05); and age, WHO clinical stages III/IV and hypercholesterolemia/normal were found to be associated with excess weight (p <0.05) independent of all other factors (Table 3). The prevalence of undernutrition decreases significantly as age increases in age 30–39 by 1.85 times (AOR = 0.54; 95% CI: 0.32–0.91) and 40–79 by 2.86 times (AOR = 0.35; 95% CI: 0.18–0.70) in comparison to age 18–29. However, WHO clinical stages III/IV was found to be significant risk factor to undernutrition (AOR = 3.54; 95% CI: 1.92–6.51).

On the otherhand, increase in age was found to be a risk factor for excess weight in age 30–39 (AOR = 3.23; 95% CI: 1.38–7.55) and 40–79 (AOR = 3.64; 95% CI: 1.47–8.98) in comparison to age 18–29. However, excess weight decreases significantly at WHO clinical stages III/IV by 3.13 times (AOR = 0.32; 95% CI: 0.10–0.98). Serum TC ≥200 mg/dL was also found to be a risk factor to excess weight (AOR = 3.94; 95% CI: 2.03–7.77). Furthermore, only older age 40–79 is found a risk factor for obesity (AOR = 0.041; 95% CI: 1.06–14.70).

**Fig 1:**
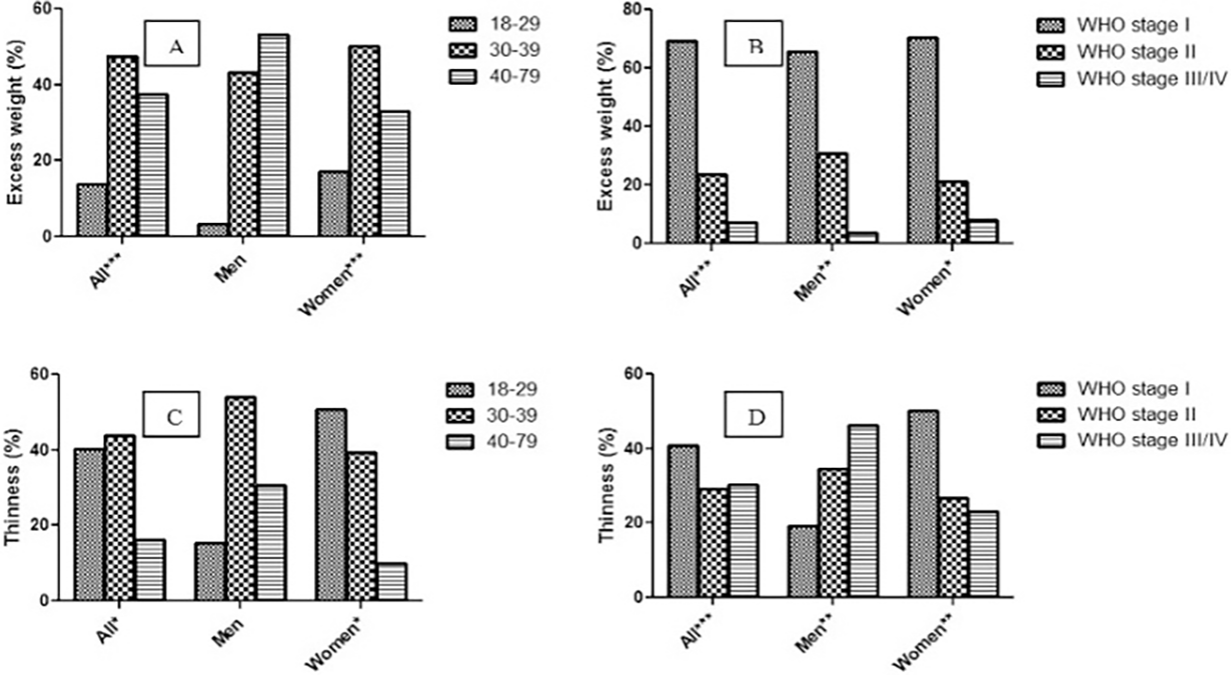
Prevalence of excess weight and thinness (undernutrition) among adults aged ≥18. The prevalences are presented by sex and age categories (A and C); and by sex and WHO clinical stages (B and D) among HIV-infected ART naïve study participants, February to September 2013, Addis Ababa, Ethiopia. ***, ** and * refer to significant increasing linear trend at p < 0.001, p<0.01 and p<0.05, respectively.

As indicated in Fig 1A, the prevalence of excess weight in age 18–29, 30–39 and 40–79 that were 13.5%, 49.2% and 37.3%, respectively, showed an increase in overall study participants (p<0.001). This significant trend was maintained in women but not men. The prevalence of excess weight decreased in overall, men and women study participants with the progress of WHO clinical stages (p<0.05, Fig 1B). On the otherhand, as indicated in Fig 1C, the prevalence of undernutrition in age 18–29, 30–39 and 40–79 that were 40.2%, 43.7% and 16.1%, respectively, showed a decrease of trend in overall study participants (p < 0.05). This trend was maintained in women but not in men. Interestingly, prevalence of undernutrition increased in men with progress of WHO clinical stages, but, it decreased in women and overall study participants with progress of WHO clinical stages (p<0.01, Fig 1D).

### Prevalences for hypercholesterolemia and hypertriglyceridemia

**Fig 2:**
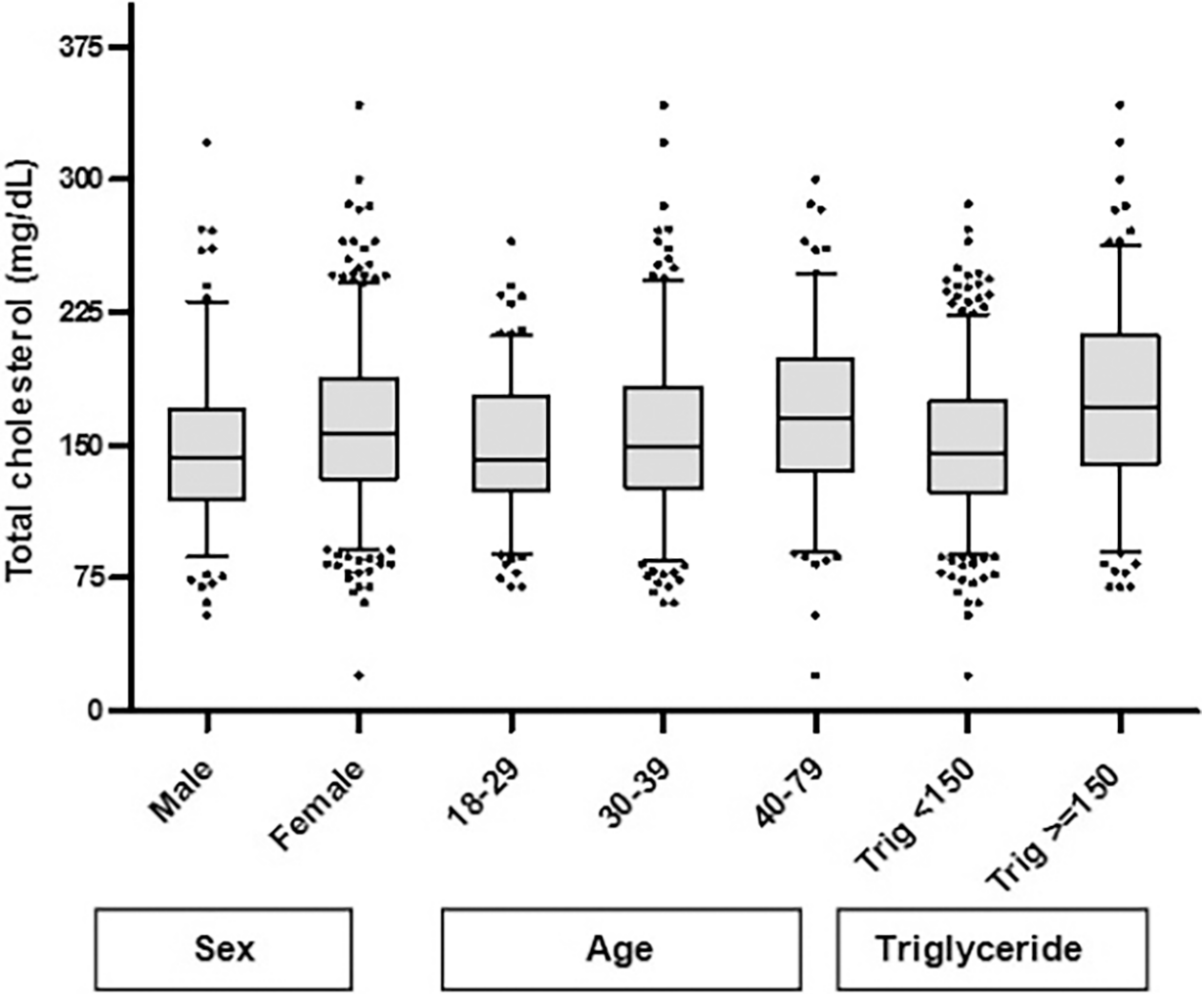
Serum total cholesterol (TC) by sex, age and triglycerides (TG) categories.

The prevalence of hypercholesterolemia was 16.6% (95% CI: 9.1–24.1) in overall study participants, 11.0% in men and 18.9% in women, which was significantly higher in women than in men (p = 0.019). Since being female, older age and higher TG were found significant risk factors (p<0.05) for increment of serum TC (Fig 2), comparison of median values of serum TC were done between these variables by Mann-Whitney and Kruskal-Wallis tests. Furthermore, the median serum TC of overall study participants was 150.0 mg/dL. The median values of serum TC were different between men and women and TG<150- and ≥150 mg/dL (p<0.001). In addition, the median values of serum TC were found significantly different between ages 18–29 and 40–79 (p<0.001), and 30–39 and 40–79 (p = 0.036). However, there was no significant difference in serum TC between ages 18–29 and 30–39 (p = 0.067). Significant Spearman correlations between CD4+ T cell count and serum TC were found (r = 0.210, p<0.001).

The prevalence of hypertriglyceridemia was 29.8% in overall study participants, 34.2% in men and 28.0% in women. There was no significant difference in prevalence of hypertriglyceridemia in men and women (p = 0.144). The median values of TG were 122.0 mg/dL in overall study participants, 128.0 mg/dL in men and 122.0 mg/dL in women. There was no significant difference between median values of TG of men and women (p = 0.113). No significant Spearman negative correlation between CD4+ T cell count and TG (r = -0.007, p>0.05).

### Risk factors for hypercholesterolemia and hypertriglyceridemia

Mann-Whitney or Kruskal-Wallis test to determine the difference in the median values of serum TC and TG were done (Table 4). Median values of serum TC were significantly different along sex, age, BMI, WHO clinical stages, being with ≥200 CD4+ T cells/mm^3^ or AIDS (<200 CD4+ T cells/mm^3^), being anemic/normal, and hypertriglyceridemia/normal. The serum TC showed an increasing trend in men in comparison to women, along increasing of age, BMI and TG. In contrast, serum TC decreased with the advancement of the disease progression as shown by WHO clinical stages, CD4+ T cell count and hemoglobin levels (p <0.01). On the otherhand, median values of TG were significantly different along age, BMI, fasting glucose level, and hypercholesterolemia/normal (p<0.01). The TG showed an increasing trend along increasing of age, BMI, fasting glucose level, serum TC (p<0.05).

**Table 4:** Median comparisons of lipid-profiles by risk factors among HIV-infected ART-naïve study participants in Addis Ababa, Ethiopia.

Variables		Total cholesterol	p value	Triglycerides	p value
		Median (IQR)		Median (IQR)	
Sex					
	Male	143.5(119.0–170.0)	<0.001	18.0(92.3–175.6)	0.133
	Female	156.0(131.0–187.0)		122.0(88.0–157.0)	
Age					
	18–29	142.0(124.5–177.0)	0.002	111.0 (77.5–150.0)	<0.001
	30–39	145.0(126.5–182.5)		121.0 (92.5–156.5)	
	40–79	165.0(135.5–199.0)		135.5 (101.3–190.5)	
BMI (kg/m^2^)					
	<18.5	144.0(120.0–176.0)	<0.001	120.0 (87.0–165.0)	<0.001
	18–24.9	149.0(127.8–179.3)		118.5 (86.8–153.3)	
	≥25	172.5(140.0–213.0)		139.0 (107.3–179.5)	
WHO clinical stage					
	Stage I	161.0(135.0–192.5)	<0.001	122.5(86.3–161.0)	0.639
	Stage II	147.0(127.0–180.8)		123.0(94.3–159.8)	
	Stage III/IV	135.0(112.0–162.5)		118.0(94.0–145.0)	
CD4+ cell count (cells/mm^3^)					
	<200	156.0(132.0–186.0)	<0.001	122.0(88.0–160.0)	0.342
	≥200	133.5(115.3–163.3)		127.5(95.4–162.8)	
Hemoglobin conc. (g/dL)					
	Non-anemic	152.0(131.0–183.0)	<0.001	122.0(88.0–160.0	0.496
	Anemic	127.0(106.5–159.5)		126.0(95.0–161.0)	
TC (mg/dL)					
	<200	-	-	117.0(86.0–150.8)	<0.001
	≥200			160.0(122.0–215.0)	
TG (mg/dL)					
	<150	146.0(123.0–175.0)		-	-
	≥150	171.5(139.0–212.3)			
Fasting glucose conc. (mg/dL)					
	<126	131.0(116.0–172.5)	0.079	154.0 (98.5–206.5)	0.033
	≥126)	NA		NA	

**Note:** Only outcome variables by the risk factors with significance difference in median values are considered. IQR = interquartile range; TC = total cholesterol; TG = triglycerides; NA = not applicable.

All the variables which showed difference in median values for serum TC (sex, age, BMI, WHO clinical stages, being HIV-positive/AIDS, being anemic/normal, and hypertriglyceridemia/normal) were significantly associated factors with hypercholesterolemia by univariate binomial logistic regression. In addition, age, fasting glucose and hypercholesterolemia/normal were also found associated with hypertriglyceridemia by univariate binomial logistic regression analysis (p<0.05, Table 5).

**Table 5:** Univariate and multivariate associations of risk factors with lipid-profiles among HIV-infected ART-naïve study participants in Addis Ababa, Ethiopia.

Variables		Total cholesterol ≥200		Triglycerides ≥150	
		COR (95% CI)	AOR (95% CI)	COR (95% CI)	AOR (95% CI)
Sex					-
	Male	1.00	1.00	1.00	
	Female	1.89(1.09–3.28)^c^	2.18(1.07–4.45)^c^	0.75(0.51–1.11)	
Age					
	18–29	1.00	1.00	1.00	1.00
	30–39	2.05(1.10–3.82)^c^	2.13(1.05–4.32)^c^	1.29(0.82–2.02)	1.15(0.68–1.95)
	40–79	3.22(1.69–6.15)^a^	2.52(1.17–5.64)^c^	1.99(1.23–3.24)^b^	1.36(0.76–2.42)
BMI (kg/m^2^)					
	<18.5	1.00	1.00	1.00	-
	18–24.9	1.08(0.52–2.24)	0.72(0.32–1.59)	0.76(0.45–1.28)	
	≥25	3.51(1.64–7.54)^a^	1.82(0.77–4.30)	1.46(0.81–2.64)	
WHO clinical stages					
	Stage I	1.00	1.00	1.00	-
	Stage II	0.60(0.36–1.00)	0.69(0.39–1.24)	0.92(0.62–1.38)	
	Stage III/IV	.20(0.07–0.56)^b^	0.36(0.11–1.11)	0.65(0.36–1.15)	
CD4+ cell count (cells/mm^3^)					
	<200	1.00	1.00	1.00	-
	≥200	0.35(0.15–0.77)^c^	0.55(0.23–1.32)	1.22(0.76–1.95)	
Hemoglobin conc. (g/dL)					
	Non-anemic	1.00	1.00	1.00	-
	Anemic	0.24(0.07–0.80)^c^	0.41(0.12–1.39)	1.20(0.68–2.12)	
TC (mg/dL)					
	<200	-	-	1.00	1.00
	≥200			3.47(.2.21–5.47)^c^	3.36(1.98–5.69)^c^
TG (mg/dL)					
	<150	1.00	1.00	-	-
	≥150	3.48(2.21–5.47)^a^	4.80(2.57–8.97)^a^		
Fasting glucose level (mg/dL)					
	<126	1.00	-	1.00	1.00
	≥126	0.81(0.27–2.39)		3.34(1.56–7.15)^b^	3.42(1.55–7.53)^b^

Note

a, b, c refers to p value <0.001, <0.01 and <0.05, respectively. CI = confidence interval; COR = crude odds ratio; AOR = adjusted odds ratio; TC = total cholesterol; TG = triglycerides. Only variables which have significant association with the outcome variable in univariate analysis are considered for multivariate analysis.

The results of multivariate binomial logistic regression analysis (Table 5) indicated significant risk factors for hypercholesterolemia (p<0.05) in this study after analyzing factors which were already significant by univariate binomial logistic regression. The independent risk factors for hypercholesterolemia were found being female (AOR = 2.18, 95% CI = 1.07–4.45) in comparison with male, age 30–39 (AOR = 2.13; 95% CI = 1.05–4.42) and 40–79 (AOR = 2.52; 95% CI = 1.17–5.64) in comparison with age 18–29, and hypertriglyceridemia (AOR = 4.80, 95% CI = 2.57–8.97) in comparison to the normal TG. In addition, diabetes (AOR = 3.42, 95% CI = 1.55–7.53) in comparison with normal fasting glucose levels, hypercholesterolemia (AOR = 3.36, 95% CI = 1.98–5.69) in comparison with normal serum TC were found to be significant independent risk factors for hypertriglyceridemia.

## Discussion

Undernutrition is more common in developing countries, where HIV patients are often not diagnosed or do not commence ART until they have advanced disease. In this study, among those who were found at AIDS stage, 53.1% were enrolled for less than a year in ART care centers for their follow up for free ART. This indicates that still there are individuals who do not get tested for HIV in fear of stigmatization and they come at late stage of the disease for test and then to treatment. This indicates that the Ministry of Health and other concerned bodies should work in more awareness creation and convincing for voluntary testing for HIV to prevent new infection.

The prevalence of undernutrition in this study population was 15.1%, which was 15.8% in men and 14.8% in women. According to the nationwide Ethiopian Demographic and Health Survey (EDHS) report of 2011 [36], the prevalence of undernutrition was 36.6% in men and 26.9% in women age 15–49 nationwide. However, the prevalence of undernutrition in Addis Ababa was 22% in men and 14.4% in women. This study indicated that undernutrition is lower in this study compared with nationwide community based EDHS report, but the prevalence of undernutrition in the community based study in EDHS is comparable in women but higher in men than this study in Addis Ababa. The prevalence of undernutrition was 3.2% in Nairobi, Kenya [37] and 5.9% in Dar es Salaam, Tanzania [38], which was lower than the prevalence of undernutrition in Addis Ababa, Ethiopia. This prevalence was comparable with the prevalence of undernutrition at Dilla University Referral Hospital (12.3%) [39]. However, the prevalence of undernutrition in women on ART in Humera Hospital was found to be 42.3% [40]. The reason for the lower prevalence of undernutrition in this ART naïve study population in comparison with those studies in HIV-infected individuals on ART could be that undernourished HIV-infected individuals has suboptimal response to HIV drugs when they start ART at CD4+ T cell count <200 cells/mm^3^ or WHO clinical stages III/IV [5, 6] so that there may not be significant improvement in their nutritional status. The other possible reasons for low prevalence of undernutrition in men in this study population were a better household food security, dietary diversity, and nutritional care and support since the study is done in Addis Ababa that has better standard of living in comparison to other parts of the country. In addition, most study participants in this study (82.8%) were with better immunological status (CD4+ T cell count ≥200 cells/mm^3^) that could protect against undernutrition [1, 4, 6].

The overall prevalence of excess weight and obesity in this study were 22.1% and 5.4%, respectively. Excess weight and obesity in this study were 17.6% and 3.6% in men, and 23.8% and 6.1% in women, respectively. According to the EDHS report, the prevalence of excess weight was 2% in men and 6% in women age 15–49 nationwide. And, the prevalences of excess weight and obesity were 12.4% and 1.6% in men, and 19.9% and 4.0% in women age 15–49, respectively [36]. This indicated that excess weight and obesity were higher in this study than the nationwide community based study in Addis Ababa. The prevalences of excess weight and obesity were 41.4% and 11.2% in Nairobi, Kenya [37] and 44.6% and 16.4% in Dar es Salaam, Tanzania [38], respectively, as reported in the respective Demographic and Health Surveys, which is higher than the prevalences in Addis Ababa, Ethiopia. In addition, the prevalence of excessive weight surpassed that of undernutrition in the overall study population (22.1% vs 15.1%). The same results were reported in other studies [29, 41]. This may be because wasting syndrome has been linked to AIDS [2] that may lead HIV-infected individuals to eat a high-calorie diet and avoid physical activity that can increase weight and lipid levels [7, 8]. The pattern of nutritional impairments in the developing world is further complicated by socio-economic changes which are taking place due to urbanization and changing lifestyles [8, 29]. This also signifies that our study is done in urban setting, Addis Ababa that is different from other dominantly rural parts of the country in socio-economics and lifestyle. Introduction of free ART service [42] left out ART naïve individuals with better median CD4+ T cell count (357 cells/mm^3^) and higher BMI in this study. This resulted in a relatively lower prevalence of undernutrition and higher prevalence of excess weight in this study in comparison to other studies. Generally, the differences in the prevalence of undernutrition and excess weight in different studies might be due to differences in the study population, socio-economic status and availability of health care services [29].

Older age and WHO clinical stages III/IV were associated significantly with undernutrition; and older age, WHO clinical stages III/IV and serum TC were associated significantly with excess weight independent of all other variables. Older age resulted in a decreasing trend in undernutrition and increasing in excess weight, and these trends were maintained only in women study subjects as reported by Kroll *et al*. [43]. Older age was also significant risk factor for obesity. The association of WHO clinical stages III/IV with undernutrition positively and excess weight negatively indicated that undernutrition is usually appeared at the advanced stages of HIV disease but not excess weight [44]. Study from Uganda showed HIV positive persons in WHO clinical stage IV often characterized by severe wasting [45] that resulted in significant decrement of excess weight. High concentration of TC was also found an independent risk factor of excess weight. Lipid abnormalities are common in ART naïve HIV-infected patients even in the absence of major host-related risk factors for dyslipidemia [46]. An increase in TC is related to higher excess body weight which is major risk factors of CVD morbidity and mortality in developing countries [47].

The results of this study indicate that demographic and HIV disease characteristics influence lipid parameters in ART naïve patients. In addition, in patients with advanced HIV infection, significant low serum TC as well as not statistically increased TG was noted before HIV therapy was initiated. The prevalence of TC hypercholesterolemia was 16.6% in overall study participants. This prevalence was higher in women (18.9%) than men (11.0%) (p = 0.019). However, the prevalence of hypertriglyceridemia was 29.8% in overall study participants and it was not significantly different between men (34.2%) and women (28.0%) (p = 0.144). Other studies in resource limited settings on ART naïve HIV-infected individuals showed that the prevalences of TC hypercholesterolemia and hypertriglyceridemia were 11.1% and 31%, respectively, in Burayu in suburb of Addis Ababa [48]; and serum TC hypercholesterolemia and hypertriglyceridemia occurred in 15.9% and 31.0%, respectively, in Hawassa [49], that are comparable to the present study. A study from Cameroon showed the prevalence of serum TC hypercholesterolemia of 24.6% in ART naive HIV-infected individuals, which is higher than the prevalence in this study [11].

There were some limitations in this study. The results may underestimate the burden of undernutrition and overestimate excess weight because we did not include patients with more severe disease presentation, such as those with cognitive impairment and immediate intensive care requirement. Since the study design was cross-sectional, it is not possible to determine the causal relationship between risk and outcome variables. Environmental factor like impact of diet has not been assessed and inclusion of HIV negative controls would have made possible comparison of biochemical changes imposed by HIV infection without the treatment. The role of specific opportunistic infections in determining the nutritional status and lipid abnormalities cannot be justified effectively because screening for opportunistic infections was not routinely done. Therefore, there is need for prospective cohort or case control studies to investigate undernutrition, excess weight and the evolution of lipid abnormalities in ART naïve HIV-infected patients. In addition, serological evidence of other concomitant infections such as hepatitis B/C, family history of dyslipidemia, co-morbid diseases such as diabetes mellitus or CVD, changes in mood, depression, factors related to lifestyle (smoking and physical inactivity) and those currently using drugs with possible interference with serum lipid levels were unaccounted for due to lack of facilities for such screening.

In conclusion, this study has provided data on the characteristics of malnutrition and lipid abnormalities of ART naïve HIV-positive patients, and associated factors. Undernutrition was prevalent in HIV-infected individuals at the start of the pandemic before the start of ART, and it is still high with the implementation of ART. However, there is an increase of overweight and obesity, and hypercholesterolemia and hypertriglyceridemia that may become a growing health problem in HIV-infected individuals. There may be a need of nutritional therapy of malnourished HIV-infected individuals. HIV-infected patients should be routinely screened for lipid disorders before commencement of ART. Individuals with higher dyslipidemia should be treated to increase survival at the time of initiating ART and then periodically through treatment follow-up to monitor any rising trends. In addition, country wide population-based prospective studies are needed to further explore the relationship between nutritional status, and lipid profile changes and immunological status of ART naïve HIV-infected patients.

## Supporting information

S1 Table Study participant demographic, socioeconomic, clinical and behavioral data collecting questionnaire. (DOC)

S2 Table Malnutrition data. (SAV)

S1 Fig Ethical clearance. (PDF)

## Abbreviations

ABCA1: ATP binding cassette subfamily A member 1
AIDS: Acquired Immune deficiency syndrome
ALERT: All African Leprosy Research and Training Centre
AOR: Adjusted odds ratio
APOA, B & C: Apolipoprotein A, B & C, respectively
ART: Antiretroviral therapy
ATP: Adult Treatment Panel/Adenosine triphosphate
BMI: Body mass index
CETP: Cholesterylester transport protein
CI: Confidence interval
COR: Crude odds ratio
CVD: Cardiovascular disease
EDHS: Ethiopian demographic and health survey
EDTA: Ethylene-diamine-tetraacetic acid
FFA: Free fatty acid
HAPCO: HIV/AIDS Prevention and Control Office
HDL-C: High density lipoprotein cholesterol
HIV: Human immunodeficiency virus
IFG: Impaired fasting glucose
IQR: Interquartile range
IRERC: Institutional Research Ethics Review committee
LDL-C: Low density lipoprotein cholesterol
LDLR: LDL receptor
LRP: Low density receptor related protein
NCEP: National Cholesterol Education Program
RCT: Reverse cholesterol transport
SNP: Single nucleotide polymorphism
TC: Total cholesterol
TG: Triglycerides
VLDL-C: Very low density lipoprotein cholesterol
WHO: World Health Organization
