# Supplementary material for: Malnutrition and lipid abnormalities in antiretroviral naïve HIV-infected adults in Addis Ababa: A cross-sectional study
Source: PLoS One. 2018 Apr 19;13(4):e0195942. doi: 10.1371/journal.pone.0195942 (PMC5908150; doi:10.1371/journal.pone.0195942)
Supplement: S1 Table — (DOC) [file pone.0195942.s001.doc]

| **Directions for filling up the enclosed questionnaire:**   - **Please mark “x” on the appropriate choice.** - **Give the details as required in the space provided.** | | | | | | | | | | |
| --- | --- | --- | --- | --- | --- | --- | --- | --- | --- | --- |
| **Sec.** | | **Questionnaire parameters** | | | | | **Coding categories** | | |  |
| **Section A: Identification** | | | | | | | | | |  |
| **A01.** | | **Hospital Card No.: _________________________________________________________** | | | | | | | |  |
| **A02.** | | **Subject ID: _______________________________________________________________** | | | | | | | |  |
| **A03.** | | **Telephone address of the study subject: ______________________________________** | | | | | | | |  |
| **A04.** | | **Date of interview** | | | | | **_______/______/________ E.C.**  **dd mm yyyy** | | |  |
| **A05.** | | Study site/facility name and address | | | | | Facility Name _______________________________ | | |  |
| **A05.** | | Visit type | | | | | Baseline 1  6 months 2  0thers________________________________ | | |  |
| **Section B: study participant profile** | | | | | | | | | | |
| **B01.** | | **Sex of the participant** | | | | | **Male 1**  **Female 2** | | |  |
| **B02.** | | **What is your birthday (age)?** | | | | | **date: _____/_____/ __________ E.C.**  **dd mm yyyy**  **age: _______________________** | | |  |
| **B03.** | | **What is your place of residence?** | | | | | **Urban 1**  **Rural 2**  **Name of district _________________________** | | |  |
| **B04.** | | **What is your level of education?** | | | | | **No education 0**  **Primary 1**  **Secondary 2**  **Tertiary 3** | | |  |
| **C05.** | | **What is your occupation?** | | | | | **Unemployed 0**  **Casual labourer 1**  **Peasant farmer 2**  **Government employee 3**  **Private employee 4**  **Self employed 5**  **other specify ________________________ 6** | | |  |
| **B06.** | | **What is your marital status?** | | | | | **Never married 0**  **Married 1**  **Separated 2**  **Divorced 3**  **widow/widower 4**  **Living with partner 5** | | |  |
| **B07** | | **What is your ethnicity?** | | | | | **Amhara 1**  **Oromo 2**  **Tigraway 3**  **Guragie 4**  **Specify other (Affar, Gamo, Hadyia, Kembata, Nuwer, Anyiwak, Sidamo, Siltie, Welaita) _______________ 5** | | |  |
| **B08.** | | **What is your status of work?** | | | | | **Working full time 1**  **Working part-time 2**  **not working due to ill health/studying 3**  **Others (specify): ________________________ 4** | | |  |
| **B09.** | | **Number of individuals in the household?___________________________** | | | | | | | |  |
| **B10.** | | **Estimated total monthly income of household? ___________________________Ethiopian Birr** | | | | | | | |  |
| **Section C: Individual’s behaviour** | | | | | | | | | |  |
| **C01.** | | **Do you do physical exercise?** | | | | | | **Never exercising 1**  **Currently exercising 2**  **Exercise in past but stopped ___yrs and ___months ago 3** | |  |
| **C02.** | | **Do you smoke cigarette?** | | | | | | **Never smoked 1**  **Currently smoking 2**  **Smoked in past but stopped ___yrs and ___months ago 3** | |  |
| **C03.** | | **If currently/ smoked in past, how many cigarettes/pipes per day do/did you usually smoke (1 pack equals 20 cigarettes)? ______________________________________________________** | | | | | | | |  |
| **C04.** | | **If you have smoking history, how long have/had you smoked? ____________________________ years** | | | | | | | |  |
| **C05.** | | **Do you drink alcohol?** | | | | | **Yes 1**  **No 2**  **Stopped now ≥ 6 months 3** | | |  |
| **C06.** | | **How often do you drink?** | | | | | **Daily 1**  **4-6 days per week 2**  **2-3 days per week 3**  **≤ 1 day in a week 4** | | |  |
| **C07.** | | **Do you use soft drugs (e.g., khat, shisha, etc)?** | | | | | **Yes 1**  **No 2**  **Stopped now ≥ 6 months 3** | | |  |
|  | | | | | | | | | | |
| **Section D: HIV clinical symptoms and test history (only filled by the clinician)** | | | | | | | | | | |
| **D01.** | | | **When did you think you are infected with the virus?** | | | | | | **date: _____/_____/ __________**  **dd mm yyyy** |  |
| **D02.** | | | **When did you know your HIV status?** | | | | | | **date: _____/_____/ __________**  **dd mm yyyy**  Name of health facility: ________________ |  |
| **D03.** | | | **Clinical symptom screen (mark “x” on all that apply)** | | | | | | |  |
|  |  |  | - **Chronic cough** - **Weight loss** - **Flu-like upper respiratory tract infection** - **Poor appetite** - **Night sweats** - **Fever >1 month** | - **Chronic diarrhea** - **Nausea/vomiting** - **Rash** - **Swelling lymph nodes** - **Numbness/tingling** | | | | | - **mental confusion** - **Stomatitis, dysphagia and/or odynophagia** - **General fatigue** - **STI symptoms** - **Others _____________________________________** |  |
| **D04.** | | | **OPPORTUNISTIC ILLNESS (MARK “X” ON ALL THAT APPLY)** | | | | | | |  |
|  |  |  | **E01. WHO STAGE 1 CONDITIONS** | | **E04. WHO STAGE 4 CONDITIONS** | | | | |  |
|  |  |  | - **Persistent generalized lymphadenopathy (PGL)** | | - **Extrapulmonary tuberculosis** | | | | |  |
|  |  |  | **E02. WHO STAGE 2 CONDITIONS** | | - **Atypical mycobacteriosis** | | | | |  |
|  |  |  | - **Minor mucocutaneous manifestations** | | - **Cryptococcosis extrapulmonary** | | | | |  |
|  |  |  | - **Weight loss <10% of body weight** | | - **Herpes simplex (mucocutaneous >1 month, visceral)** | | | | |  |
|  |  |  | - **Herpes zoster (shingles)** | | - **HIV-related encephalopathy** | | | | |  |
|  |  |  | - **Recurrent urtis** | | - **Lymphoma** | | | | |  |
|  |  |  | **E03. WHO STAGE 3 CONDITIONS** | | - **Mycosis, disseminated (histoplasma, coccidioides)** | | | | |  |
|  |  |  | - **Oral candidiasis** | | - **Salmonella septicaemia, non-typhoid** | | | | |  |
|  |  |  | - **Oral hairy leukoplakia** | | - **HIV wasting syndrome** | | | | |  |
|  |  |  | - **Unexplained chronic diarrhea (>1 month)** | | - **Candidiasis (esophagus, trachea, bronchi or lungs)** | | | | |  |
|  |  |  | - **Unexplained prolonged fever (>1 month)** | | - **Cryptosporidiosis with diarrhea (>1 month duration)** | | | | |  |
|  |  |  | - **Weight loss >10% of body weight** | | - **Cytomegalovirus (CMV) disease (other than liver, spleen, lymphnodes)** | | | | |  |
|  |  |  | - **Recurrent bacterial pneumonia** | | - **Kaposi’s sarcoma** | | | | |  |
|  |  |  | - **Other severe bacterial infections (*i.e.,* pyomyositis)** | | - **Progressive multifocal leukoencephalopathy (PML)** - **Pneumocystis carinii pneumonia (PCP)** | | | | |  |
|  |  |  | - **Pulmonary tuberculosis** | | - **Toxoplasmosis of the brain** | | | | |  |
| **D05.** | | | **WHO staging based on opportunistic illness** | | **WHO stage 1 1**  **WHO stage 2 2**  **WHO stage 3 3**  **WHO stage 4 4** | | | | |  |
| **D06-1.** | | | **Did you take any prescription drug for opportunistic infections?** | | **Yes 1**  **No 2** | | | | |  |
| **D06-2.** | | | **If yes for D06-1, list any medications, nutrition supplements or vitamins that you are currently taking.**  ________________________________________________________________________________________________________ | | | | | | |  |
| **Section E. Anthropometric and other measurements** | | | | | | | | | |  |
| **E01.** | **Height** | | | | **__________________ (cm)** | | | | |  |
| **E02.** | **Weight** | | | | **___________________ (kg)** | | | | |  |
| **E03.** | **Blood pressure** | | | | **____________ (S/D mmHg)** | | | | |  |
| **E04.** | **Heart rate** | | | | **__________________ (B/M)** | | | | |  |
| **E05.** | **Respiratory rate** | | | | **__________________ (R/M)** | | | | |  |
| **E06.** | **Haemoglobin level** | | | | **__________________ (g/dL)** | | | | |  |
| **E07.** | **CD4 count** | | | | **______________(cells/mm^3^)** | | | | |  |
| **E08.** | **Glucose level** | | | | **________________ (mg/dL)** | | | | |  |
| **E09.** | **Albumin** | | | | **_______________ (mmol/L)** | | | | |  |
| **E10.** | **Total cholesterol** | | | | **________________ (mg/dL)** | | | | |  |
| **E11.** | **LDL cholesterol** | | | | **________________ (mg/dL)** | | | | |  |
| **E12.** | **HDL cholesterol** | | | | **________________ (mg/dL)** | | | | |  |
| **Filled by:**  **Date : _____/_____/ __________**  **Interviewer's Name: _______________________**  **Interviewer’s final assessment: ________­­­­­­­­­­­­­­­­­­­­­­­­­­­­­­­­­­­­­­­­­­­­­­­­­­­­­_______________________________**  **Interviewer's Signature: _____________________________________________** | | | | | | **Checked by:**  **Date : _____/_____/ __________**  **Supervisor's Name: _______________________**  **Supervisor’s final assessment_____________________**  **Supervisor's Signature:___________________________** | | | | |
