# Supplementary material for: Malnutrition and lipid abnormalities in antiretroviral naïve HIV-infected adults in Addis Ababa: A cross-sectional study
Source: PLoS One. 2018 Apr 19;13(4):e0195942. doi: 10.1371/journal.pone.0195942 (PMC5908150; doi:10.1371/journal.pone.0195942)
Supplement: S1 Fig — (PDF) [file pone.0195942.s003.pdf]

በኢትዮጵያ ፌዴራላዊ ዴሞክራሲያዊ ሪፐብሊክ
የሳይንስና ቴክኖሎጂ ሚኒስቴር

The Federal Democratic Republic of Ethiopia
Ministry of Science and Technology

ቁጥር 3.10/004/2015
Ref. No.
ቀን 26-03-2015
Date

To: Armuer Hansen Research Institute
Addis Ababa

Re: Role of HLA Polymorphism in driving HIV Variation and Influencing Disease Progress in HIV Infected Ethiopians in Four Hospitals Addis Ababa, Ethiopia

Dear Sir/Madam /Mr./Mrs./Dr.

We are writing this letter in reference to your renewal request letter dated march 10, 2015.

After having in depth review of your request, the National Research Ethics Review Committee has accepted your renewal request for one year from March 26, 2015 to March 25, 2016

This is, therefore, to notify that the ethical approval is renewed and your group can proceed in accordance to the latest approved document. Please ensure that you submit a biannual report and an annual renewal application 30 days prior to expire date. We are confident that you as PI of the project and your esteemed organization will monitor the ethical implication of the project as it is stipulated in the latest approved document.

With regards,

Yohannes Sitotaw
Secretary of NRERC

CC: _ Chairperson, NRERC

_ **Mr. Melaku Adale (PI)**

ማነጋገር ቢያስፈልግዎ
You may Contact

ፖ.ሳ.ቁ. **P.O.Box 2490**
አዲስ አበባ ኢትዮጵያ **Addis Ababa, Ethiopia**
E-mail most@ethionet.et
ስልክ **Tel. 251-011-4-674353**
Web site:-http://www.most.gov.et
ፋክስ **Fax +251-011-4-66 02 41**
